# Using anti-poly(ethylene glycol) bioparticles for the quantitation of PEGylated nanoparticles

**DOI:** 10.1038/srep39119

**Published:** 2016-12-19

**Authors:** Yuan-Chin Hsieh, Ta-Chun Cheng, Hsin-Ell Wang, Jia-Je Li, Wen-Wei Lin, Chien-Chiao Huang, Chih-Hung Chuang, Yeng-Tseng Wang, Jaw-Yuan Wang, Steve R. Roffler, Kuo-Hsiang Chuang, Tian-Lu Cheng

**Affiliations:** 1 Graduate Institute of Medicine, College of Medicine, Kaohsiung Medical University, 100 Shih-Chuan first Road, Kaohsiung 80708, Taiwan; 2 Center for Biomarkers and Biotech Drugs, Kaohsiung Medical University, 100 Shih-Chuan First Road, Kaohsiung 80708, Taiwan; 3 Department of Biomedical Imaging and Radiological Sciences and Institute of Microbiology and Immunology, National Yang-Ming University, Taipei, Taiwan; 4 Institute of Biomedical Sciences, National Sun Yat-Sen University, Kaohsiung, Taiwan; 5 Department of Medical Laboratory Science and Biotechnology, Kaohsiung Medical University, 100 Shih-Chuan first Road, Kaohsiung 80708, Taiwan; 6 Department of Biochemistry, College of Medicine, Kaohsiung Medical University, 100 Shih-Chuan First Road, Kaohsiung 80708, Taiwan; 7 Department of Surgery, Faculty of Medicine, College of Medicine, Kaohsiung Medical University, 100 Shih-Chuan first Road, Kaohsiung 80708, Taiwan; 8 Institute of Biomedical Sciences, Academia Sinica, 128 Academia Road, Section 2, Taipei 11529, Taiwan; 9 Graduate Institute of Pharmacognosy, Taipei Medical University, 250 Wuxing Street, Taipei 11031, Taiwan; 10 Ph.D. Program for Clinical Drug Discovery from Botanical Herbs, Taipei Medical University, 250 Wuxing Street, Taipei 11031, Taiwan; 11 Department of Biomedical Science and Environmental Biology, Kaohsiung Medical University, 100 Shih-Chuan First Road, Kaohsiung 80708, Taiwan

## Abstract

Attachment of polyethylene glycol (PEG) molecules to nanoparticles (PEGylation) is a widely-used method to improve the stability, biocompatibility and half-life of nanomedicines. However, the evaluation of the PEGylated nanomedicine pharmacokinetics (PK) requires the decomposition of particles and purification of lead compounds before analysis by high performance liquid chromatography (HPLC), mass spectrometry, etc. Therefore, a method to directly quantify un-decomposed PEGylated nanoparticles is needed. In this study, we developed anti-PEG bioparticles and combined them with anti-PEG antibodies to generate a quantitative enzyme-linked immunosorbent assay (ELISA) for direct measurement of PEGylated nanoparticles without compound purification. The anti-PEG bioparticles quantitative ELISA directly quantify PEG-quantum dots (PEG-QD), PEG-stabilizing super-paramagnetic iron oxide (PEG-SPIO), Lipo-Dox and PEGASYS and the detection limits were 0.01 nM, 0.1 nM, 15.63 ng/mL and 0.48 ng/mL, respectively. Furthermore, this anti-PEG bioparticle-based ELISA tolerated samples containing up to 10% mouse or human serum. There was no significant difference in pharmacokinetic studies of radiolabeled PEG-nanoparticles (Nano-X-^111^In) through anti-PEG bioparticle-based ELISA and a traditional gamma counter. These results suggest that the anti-PEG bioparticle-based ELISA may provide a direct and effective method for the quantitation of any whole PEGylated nanoparticles without sample preparation.

PEGylation of nanoparticles may improve their biocompatibility, reduce immunogenicity and enhance their half-life in the human body. PEGylated nanoparticles are widely used and have been developed into various types of nanomedicine. For example, PEG-modified liposomal doxorubicin (Caelyx and Lipo-Dox) has been used to treat ovarian, breast carcinomas and Kaposi’s sarcoma^1^,^2^. PEGylated Interferon (Pegasys^3^,^4^, PEG-Intron^5^) was employed as a long-term therapeutic agent for hepatitis C. Several PEGylated polymeric micelle formulations, such as Paclitaxel and Cisplatin, are currently in phase I/II clinical trials for treatment of stomach cancer and solid tumors^6^,^7^. PEG-modified imaging nanoparticles, such as quantum dots (QD)^8^ and clinically approved super-paramagnetic iron oxide (PEG-SPIO)^9^ have also been used to track the localization of tumors by optical or MR imaging system. An effective method to measure the pharmacokinetics of PEG-modified nanoparticles is needed for these various types of PEGylated nanomedicine and will also be important for both drug-development and clinical applications.

To date, several approaches have been proposed to measure the concentration of PEGylated nanoparticles. However, current methods have limitations. For example, radioactivity-based pharmacokinetics study is currently the most sensitive method for the measurement of PEG-liposomes or PEG-micelles through determination of incorporated radioactivity. But radioisotope-incorporation creates radio-hazards and needs a dedicated and licensed facility. High-performance liquid chromatography (HPLC) is the most common method for pharmacokinetics studies of PEGylated nanoparticles. For instance, samples of PEG-liposomes or PEG-micelles, usually in serum, have to undergo protein precipitation and active drug extraction by decomposing particles^10^ before HPLC analysis. This preparation breaks the particles and results in some deviation in the measurement of PEGylated nanoparticles. For solid PEGylated nanoparticles, such as PEG-SPIO and PEG-gold nanoparticles, inductively-coupled plasma mass spectrometry (ICP-MS) can be used to quantify and determine the nanoparticles kinetics. But, PEG-SPIO or PEG-gold nanoparticles need to be dissolved by nitric acid or Aqua Regia before ICP-MS analysis^11^. This procedure also destroys the structure of particles. Furthermore, serum also interferes with the detection ability of ICP-MS^12^. In short, current methods require the decomposition of PEGylated nanoparticles before evaluating the pharmacokinetics. They can determine the kinetics of the lead compound but not whole PEGylated nanoparticle, and may therefore result in miscalculation of the metabolism and kinetics of PEGylated nanoparticles. Based-on such shortcomings, development of a simple, sensitive and universal method to directly measure the concentrations of whole PEGylated nanoparticles is very important for pharmacological studies.

Based on this rationale, in this study we attempted to develop a method for direct measurement of PEGylated nanoparticles without compound purification. We expressed anti-PEG antibody Fab on the cell surface to form anti-PEG bioparticles and combined it with anti-PEG antibodies to generate a quantitative ELISA (anti-PEG bioparticle-based ELISA) for direct measurement of PEGylated nanoparticles without compound purification (Fig. 1). We checked the membrane expression and functions of the anti-PEG bioparticles by fluorescence conjugated anti-tag antibodies and PEGylated probes. We then examined the functionality of the anti-PEG bioparticle-based ELISA by fixing with 1% paraformaldehyde and further investigated the detection limit of the anti-PEG bioparticle-based ELISA. We also examined whether the anti-PEG bioparticle-based ELISA could tolerate samples in human or mouse serum. We used a radiolabeled PEG-nanoparticle (Nano-X-^111^In) to compare the detection ability of the anti-PEG bioparticle-based ELSIA and a traditional radioactivity-based gamma counter in pharmacokinetic studies. The results suggest that the anti-PEG bioparticle-based ELISA may provide a direct and effective method for the quantitation of any whole PEGylated nanoparticles without sample preparations.

## Results

### Characterization of anti-PEG bioparticles

**Figure 2:**
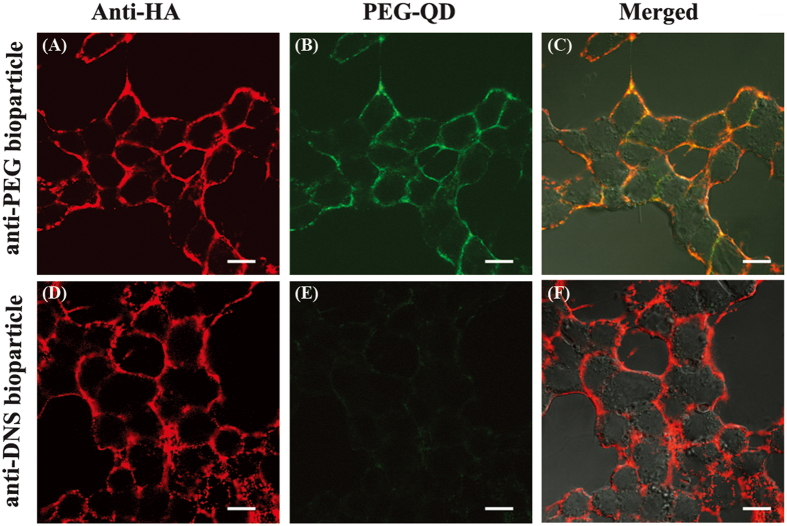
Characterization of anti-PEG bioparticles. Anti-PEG bioparticles (top row) and anti-DNS control bioparticles (bottom row) were stained with α-HA antibody (red fluorescence) and PEG-QD (green fluorescence). Bioparticles were observed with a digital confocal microscope. Merged images are shown. Scale bars in this figure correspond to 10 μm.

The properties of anti-PEG or anti-Dansyl (DNS) bioparticles were determined by immunofluorescence staining with mouse anti-HA tag antibodies and PE-conjugated goat anti-mouse IgG Fc antibody. Figure 2A,D shows that PE signal (red florescence) was accumulated on both the surfaces in the anti-PEG bioparticle and anti-DNS bioparticle groups. This result indicates that both the anti-PEG bioparticles and anti-(DNS) bioparticles were successfully established. In order to examine the specific binding ability of anti-PEG bioparticles, the PEG-QD were incubated to the anti-PEG bioparticles and anti-Dansyl (DNS) bioparticles. Figure 2B,E show that QD signals (green florescence) only accumulated on the surface of anti-PEG bioparticles, and not the control anti-DNS bioparticles. The merged results of anti-PEG bioparticles and PEG-QD were shown as a co-localized feature (Fig. 2C), but, the anti-DNS bioparticles only showed the antibody expression (Fig. 2F). These results demonstrate that anti-PEG antibody Fab was successfully expressed on the cell surface to form the anti-PEG bioparticles and PEGylated nanoparticles can be specifically trapped at the anti-PEG bioparticles.

### Assessment of sensitivity with paraformaldehyde fixation in the anti-PEG bioparticle-based sandwich ELISA

**Figure 3:**
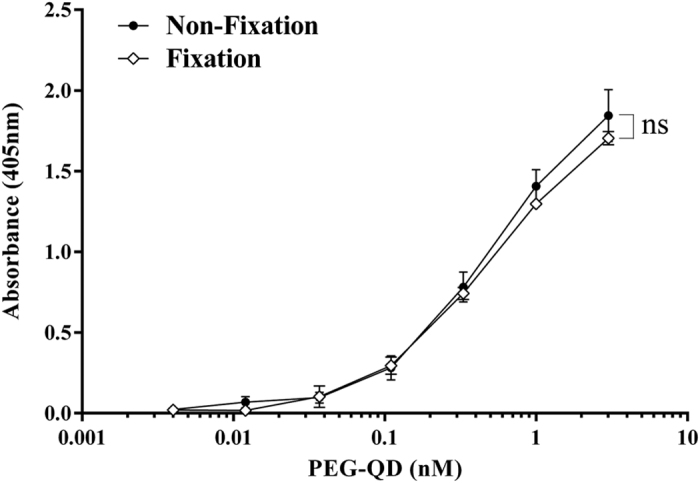
Measurement of the concentration of PEG-QD nanoparticles in fixed anti-PEG bioparticle-based sandwich ELISA. Sandwich ELISA in which anti-PEG bioparticle/AGP4-biotin was employed as the capture/detection reagents to measure PEG-QD in the paraformaldehyde fixation (♢) and non-fixation (●) anti-PEG bioparticle. Representative data from three independent experiments are shown. Data represents mean ± SD. Statistical analysis was performed by independent t-test (P = 0.9044). ns, no significant difference.

The detection sensitivity of paraformaldehyde fixed or non-fixed anti-PEG-bioparticle-based ELISA was performed by measuring the PEG-NPs. PEGylated nanoprobes (PEG-QD) were added to anti-PEG bioparticle-coated 96-well plates. Then, anti-PEG-Biotin antibodies were used as detection antibodies. Figure 3 shows that the detection limit of PEG-QD was consistent in fixed or non-fixed anti-PEG bioparticle-based ELISA. The results show that the sensitivity of anti-PEG bioparticle-based ELISA could not be reduced with the fixation of paraformaldehyde.

### Quantitation of PEGylated nanoparticles by anti-PEG bioparticle-based ELISA

**Figure 4:**
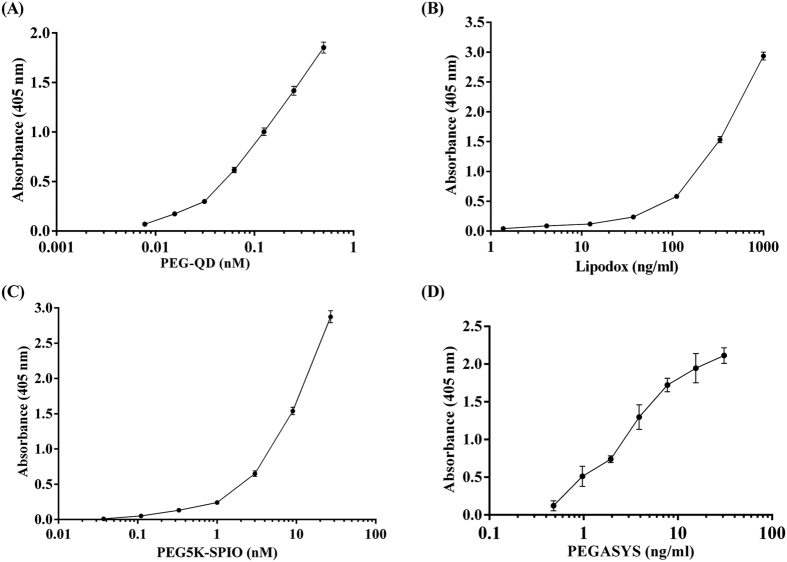
Quantification of PEGylated nanomolecules by anti-PEG bioparticle-based sandwich ELISA. Anti-PEG bioparticles acted as the capture reagents and AGP4-biotin as the detection antibody to measure the concentration of (**A**) PEG-QD (**B**) Lipodox, (**C**) PEG5K-SPIO and (**D**) PEGASYS. Representative data from three independent experiments are shown. Data represents mean ± SD.

The detection limit of anti-PEG bioparticle-based ELISA was measured by PEGylated nanomolecules. PEGylated nanomolecules (Lipo-dox, PEG-QD, PEG-SPIO and PEGASYS) were added to anti-PEG bioparticle-coated 96-well plates. Then, anti-PEG-Biotin antibodies were used as detecting antibodies. As shown in Fig. 4A–D, the concentrations of PEGylated nanoparticles can be measured by anti-PEG bioparticle-based ELSA and the detection limit of PEG-QD, PEG-SPIO, Lipo-dox and PEGASYS are 0.01, 0.1 nM, 15.63 and 0.48 ng/mL, respectively. These results indicate that we successfully established an anti-PEG bioparticle-based ELISA with high sensitivity for PEGylated nanoparticle quantitation.

### Tolerance to serum effect of the anti-PEG bioparticle-based ELISA

**Figure 5:**
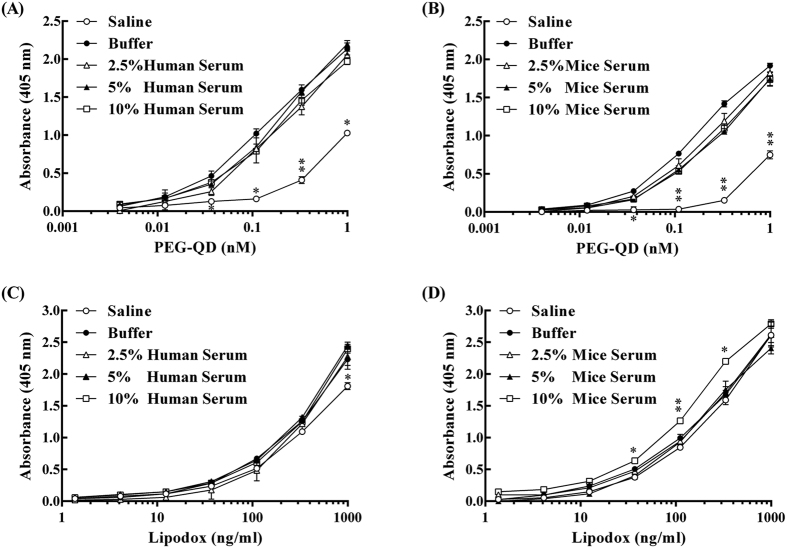
Effect of serum in the anti-PEG bioparticle-based sandwich ELISA. Sandwich ELISA in which anti-PEG bioparticle/AGP4-biotin were employed as the capture/detection reagents to measure Lipo-Dox and PEG-QD in the presence of saline (○), buffer: saline containing 2% (wt/vol) skim milk (●) or 2% (wt/vol) skim milk containing 2.5% (v/v) serum (△), 5% (v/v) serum (▲), or 10% (v/v) serum (□), respectively. Human serum (**A**) (**D**) and mouse serum (**B**) (**C**) were investigated. Representative data from three independent experiments are shown. Data represents mean ± SD. Statistical analysis was performed by multiple t-test. *P value < 0.05; **P value < 0.0001 as compared to buffer group (●).

The effect of serum on the sensitivity of the anti-PEG bioparticle particle-based ELISA was examined by measuring concentrations of PEG-QD or Lipo-Dox under human or mouse serum-containing conditions. The seral diluted PEG-QD and Lipodox were prepared in 2.5%, 5% or 10% human or mouse serum and were then added to an anti-PEG bioparticle-coated 96-well plate. Anti-PEG-Biotin antibodies were used as detection antibodies. For measurement of PEG-QD, the detection limit was 0.01 nM. Similar results were also obtained in the measurement of Lipo-Dox. The detection limit was as low as 15.63 ng/mL of Lipo-Dox the detection profiles were also almost the same in the groups containing different percentages of human (Fig. 5A,C) or mouse serum (Fig. 5B,D). We also noted lower absorbance signals in the saline group, without skim milk or serum, when measuring PEG-QD or Lipo-Dox. This phenomenon demonstrates that skim milk or serum protein could enhance and stabilize the antibody binding in anti-PEG bioparticle-based ELISA. These results indicate that the anti-PEG bioparticle-based ELSIA is serum tolerant and the detection sensitivity can be enhanced by a supporting or serum protein.

### Comparison of the detection ability of anti-PEG bioparticle-based ELISA and radioactivity assay for pharmacokinetic study of Nano-X-^111^In

**Figure 6:**
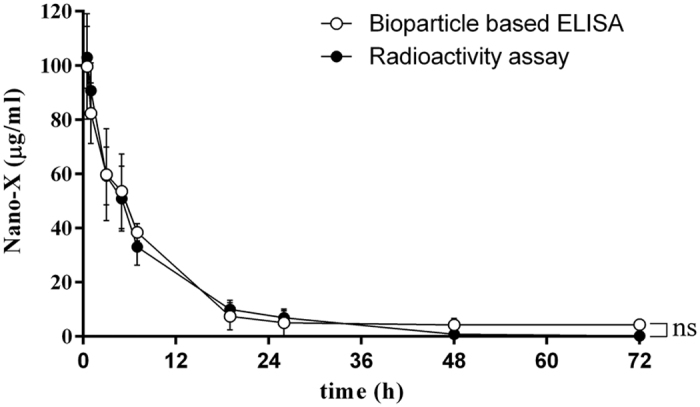
Pharmacokinetics of Nano-X-^111^In in mice. BALB/c mice (n = 8) were intravenously injected with 20 μCi of Nano-X-111In. The concentration of Nano-X-^111^In was measured by the anti-PEG bioparticle-based ELISA (○). The radioactivity of Nano-X-^111^In in the serum sample was directly measured by a gamma counter (●). Data represents mean ± SD. Statistical analysis was performed by independent t-test (P = 0.9994). ns, no significant difference.

The detection ability of anti-PEG bioparticle-based ELISA and traditional radioactivity-based pharmacokinetics study were compared. Twenty microcurie radiolabeled PEG-nanoparticles (Nano-X-^111^In) were intravenously injected into Balb/c mice. Mouse serum was harvested at different times and the concentration of Nano-X-^111^In was determined by anti-PEG bioparticle-based ELISA or a gamma counter. Figure 6 shows that the pharmacokinetic results of Nano-X-^111^In which was determined by anti-PEG bioparticle-based ELISA (Half-Life = 4.5 hour) are similar to the gamma counter method (Half-Life = 3.8 hour). These results indicate that the anti-PEG bioparticle-based ELSIA is a highly sensitive platform, comparable to the radioactivity-based method, for quantifying the PEGylated nanoparticles in pharmacokinetic studies.

## Discussion

In this study, we have successfully established an anti-PEG bioparticle-based ELISA platform for the quantitation of whole PEGylated nanoparticles. The anti-PEG bioparticles recognize the PEG backbone (-O-CH_2_-CH_2_) on the surface of PEGylated nanoparticles. The anti-PEG bioparticles were able to combine with anti-PEG antibodies to directly quantify Lipo-Dox, PEG-SPIO, PEG-QD and PEGASYS without particle breaks and lead compound purification. Furthermore, this anti-PEG bioparticle-based ELISA could tolerate the serum effect and 1% paraformaldehyde fix for commercialization. Furthermore, the results of pharmacokinetic studies on Nano-X-^111^In through anti-PEG bioparticle-based ELISA are comparable to the traditional radioactivity-based method. Those results demonstrate that the anti-PEG bioparticle-based ELISA is a powerful tool to measure any PEGylated nanoparticles and to help accelerate the development of clinical and preclinical nanomedicines.

A universal platform is needed to quantify various types of PEGylated nanoparticles. PEGylation, PEG incorporation, is approved by the US Food and Drug Administration and is a widely-used method to improve the stability, biocompatibility and half-life of nanomedicines^13^,^14^. But, a corresponding method should be used to determine the kinetics of each type of PEGylated nanomedicine. Traditional approaches for estimating the PK, such as HPLC have been utilized to analyze the liposomal drugs^15^ and ICP-MS is used to analyze the solid nanoparticles^16^,^17^. Our anti-PEG bioparticles could recognize the backbone repeat (-CH_2_-CH_2_-O-) of PEG on the surface of nanoparticles. In our previous report, we reported that PEG-biocapture particle-based ELISA can be used to determine the kinetics of various lengths of PEG, such as free PEG (M.W. 1000, 2000, 5000)^18^. The anti-PEG antibodies also can measure the liner PEG modified interferon alfa-2b or branch PEG modified interferon alfa-2a^19^. In this study, the anti-PEG bioparticle-based ELISA was used to assess the concentrations of Lipo-Dox with PEG2000, and PEG-Qdot with PEG5000. The results indicated that this anti-PEG bioparticle-based ELISA is a total solution for the analysis of PK various types of PEGylated nanoparticles in a single approach. It may also be a useful method for estimating PK *in vivo* thus accelerating the development of PEGylated nanomedicine.

Development of a quantitative platform that tolerates serum-containing samples is important for *in vivo* pharmacokinetic studies of nanomedicines. Most samples for analysis in pharmacokinetic studies are in serum. However, serum contains multiple substances that cause serum interference in kinetic analysis, including serum proteins, non-specific antibodies, fatty acids, bile salts, ligands, and steroids^20^,^21^. Most sample preparation procedures are to reduce the effect of serum. For example, Caroline reported that using acids or heat to remove the nonspecific-protein from serum before sample injection, can reduce the nonspecific signal in liquid chromatography-mass spectrometry (LC-MS) analysis^22^,^23^. Remsberg reported that for the measurement of kinetics of anti-cancer mTOR inhibitor drug (Ridaforolimus-DSPE-PEG2000 Micellar) in rats, the inhibitors should be extracted from serum by ethyl acetate before HPLC analysis^24^. Although sample preparations and extractions are common in current analytic methods (LC-MS, HPLC, etc.), the recovery rate may directly affect the results of kinetic studies. Because the PEGylated nanoparticles break, the kinetics of whole PEGylated nanoparticles cannot be determined in kinetic studies. Our previous studies also found that the human serum affected the detection sensitivity of PEGylated interferon (Pegasys) in traditional sandwich ELISA^25^. As mentioned above, multiple-step extraction and serum may affect the sensitivity and accuracy for determining the pharmacokinetics of PEGylated nanoparticles. In our study, the concentration of PEG-NPs in serum samples could be detected with anti-PEG bioparticle-based ELISA without any extraction step. Furthermore, the anti-PEG bioparticle-based ELISA, with anti-PEG bioparticles expressing anti-PEG antibody Fab on the cell surface, could tolerate up to 10% human or mouse serum as quantitating PEG-Lipo-Dox or PEG-QD. Moreover, serum proteins or supporting proteins could stabilize the antibody binding and enhance detection sensitivity up to eight-fold in the anti-PEG bioparticle-based ELISA. These results indicate that the anti-PEG bioparticle-based ELISA system can determine the concentration of the PEGylated nanoparticles without sample extraction and tolerance to serum interference. It may provide an easy handing platform to quantitate whole PEGylated nanoparticles for pharmacokinetic studies *in vivo*.

Anti-PEG bioparticle-based ELISA is an economically competitive platform for quantitation of PEGylated nanomedicines. Kinetics analysis using ELISA requires high quality antibodies. Traditionally antibodies are usually harvested from ascites of living animals and purified by a protein A/G column. However, this approach causes pain in animals and lower antibody yield in unstable antibody purification buffer conditions^26^,^27^,^28^. To reduce the pain in the animal, European countries have also established guidelines to restrict or prohibit ascites production in rodents^29^,^30^,^31^. Several substitutable approaches have been developed to produce antibodies. For example, Hendriken *et al*. reported antibodies that were produced *in vitro* by a bioreactor^32^. Glassy and Mariani also indicated that antibodies could be produced and harvested from the serum substitute supplements containing medium of hybridoma^33^,^34^. But, the expensive and time-consuming process of purification of antibodies is also required in those *in vitro* antibody producing methods. The PEG-bioparticle is an anti-PEG antibody Fab expressed on the cell surface with uniform orientation which could sensitively recognize the PEG molecule^35^. The antibodies are automatically produced as cell proliferation. In addition, antibody purification is not necessary in this anti-PEG bioparticle producing system and the anti-PEG bioparticles can be applied to an ELSIA plate as a capture layer to detect the concentration of various PEG-NPs. This bioparticle-based strategy should be combined with the appropriate cell-coating platform for the scale plating system. However, a factory-scale cell-coated product-line should be established. We believe the anti-PEG bioparticle-based strategy may provide an economic and convenient approach for the manufacture of a commercial ELISA kit.

## Conclusion

PEGylation is still the most useful technique in nanomedicine for enhancement of bioavailability and therapeutic efficacy^36^. Current pharmacalkinetic studies of PEGylated nanoparticles are limited by radioisotope-incorporation methods or particle breaks^15^. The anti-PEG bioparticle-based ELISA provides several advantages: (1) the property of a PEG backbone dependent on anti-PEG bioparticles allows wide use in diverse PEGylated nanomedicine; (2) the orientation of anti-PEG bioparticles is directed outwards to improve detection sensitivity; (3) the anti-PEG bioparticles are auto-reproduction which reduces the cost compared to traditional methods of antibody purification; (4) anti-PEG bioparticles could be fixed with paraformaldehyde that make it possible to commercialize; (5) the tolerance to serum makes the anti-PEG bioparticles suitable to assess any PEGylated nanomedicine in a serum sample; (6) anti-PEG bioparticle-based ELISA could quantitate whole PEGylated nanoparticles without breaking the particles. Based on these features, we believe that the anti-PEG bioparticle-based platform meets a need in the development of novel nanomedicine and has a profound impact on the present quantitating technique of nanomedicines.

## Materials and Methods

### Animals and cells

Female BALB/c mice were purchased from the National Laboratory Animal Center, Taipei, Taiwan. All animal experiments were performed in accordance with institutional guidelines and were approved by the Animal Care and Use Committee of the Kaohsiung Medical University. BALB/3T3 cells (American Type Culture Collection, Manassas, VA) were grown in Dulbecco’s modified Eagle’s medium (DMEM) (Sigma-Aldrich, St. Louis, MO) containing 10% bovine calf serum (BCS) (HyClone) with 100 units/mL penicillin and streptomycin (Invitrogen, Calsbad, CA), at 37 °C in a humidified atmosphere of 5% CO_2_. The generation of BALB/3T3 cells which expressed anti PEG-bioparticles (anti-PEG antibody Fab) or control anti-DNS-bioparticles (anti-DNS antibody Fab) was as described previously^37^.

### Reagents

PEG-Qdot 525 (Qdot 525 ITK amino (PEG) quantum dots) was purchased from Invitrogen. Lipo-Dox was provided by Taiwan Tung Yang Biopharm Company Ltd. The synthesis of Nano-X-^111^In was as previously described^38^. PEG-SPIO was purchased from Genovis (Lund, Sweden). Pegsays were from Roche (NJ, USA).

### Characterization of anti-PEG bioparticles by confocal microscopy

BALB/3T3 cells (5 × 10^5^) which expressed anti-PEG antibody Fab or control anti-DNS antibody Fab^35^ were grown on 10 μg/mL poly-L-lysine coat 18 mm cover glass in culture medium at 37 °C in a humidified atmosphere of 5% CO_2_ for 24 hours. Cells were washed with DMEM once and Tris-buffered saline (TBS) twice, and incubated with 1% paraformaldehyde for 3 minutes at room temperature. The slices were washed with TBS 3 times and blocked with TBS containing 3% bovine serum albumin (BSA) for 1 hour at room temperature. The slices were washed with TBS 3 times and stained with anti-HA epitope antibody (1.5 mg/ml, 200x dilute) for 45 min at room temperature. The slices were washed with TBS 3 times and stained with Goat anti-mouse IgG-Fcγ-PE (0.5 mg/ml) for 45 min at room temperature. The slices were washed with TBS 3 times and stained with PEG-Qdot 525 4 nM for 45 min at room temperature. After extensive washing, the endocytic activity of the receptors was recorded with a confocal microscope (LSM 510 META NLO DuoScan, Carl Zeiss).

### Measurement of the concentration of PEG-QD nanoparticles by fixed anti-PEG bioparticle-based sandwich ELISA

BALB/3T3 cells which expressed anti-PEG antibody Fab (2 × 105 cells/well) were grown in 96-well plates (Nalge Nunc International, Roskilde, Denmark) in culture medium for 24 hours. Plates were washed with DMEM twice and saline once. The fixation group was treated with 1% paraformaldehyde for 3 min at room temperature, and washed with saline 3 times then blocked with saline containing 2% skim milk for 1 hour at 37 °C. The control group was incubated in DMEM buffer at 37 °C. Graded concentrations of PEG-Qdot 525 were diluted with saline containing 2% skim milk, and added to the wells (50 μL/well, n = 3 per group) at room temperature for 1 hour. After being washed, the cells were sequentially incubated with biotinylated AGP4 (0.25 μg/well) and streptavidin-conjugated horseradish peroxidase (streptavidin-HRP, 50 ng/well). The plates were washed with saline, and bound peroxidase was measured by adding 150 μL/well ABTS solution [0.4 g/mL, 2′-azinobis (3-ethylbenzthiazoline-6-sulfonic acid) (ABTS; Sigma-Aldrich), 0.003% H_2_O_2_, and 100 mM phosphate-citrate, pH = 4.0] for 30 min at room temperature. The experiment was repeated three times, independently.

### Anti-PEG bioparticle-based sandwich ELISA

BALB/3T3 cells which expressed anti-PEG antibody Fab or anti-DNS antibody Fab (2 × 10^5^ cells/well) were grown in 96-well plates (Nalge Nunc International, Roskilde, Denmark) in culture medium for 24 hours. Plates were washed with DMEM twice and saline once. Cell-coated plates were treated with 1% paraformaldehyde for 3 min at room temperature, and washed with saline 3 times then blocked with saline containing 2% skim milk for 1 h at 37 °C. Graded concentrations of PEG-Qdot 525, Lipo-Dox, PEG-SPIO, Pegasys, PEG-micelle and Nano-X-^111^In were diluted with saline containing 2% skim milk, and added to the wells (50 μL/well, n = 3 per group) at room temperature for 1 hour. After being washed, the cells were sequentially incubated with biotinylated AGP4 (0.25 μg/well) and streptavidin-conjugated horseradish peroxidase (streptavidin-HRP, 50 ng/well). The plates were washed with saline, and bound peroxidase was measured by adding 150 μL/well ABTS solution [0.4 g/mL, 2′-azinobis (3-ethylbenzthiazoline-6-sulfonic acid) (ABTS; Sigma-Aldrich), 0.003% H_2_O_2_, and 100 mM phosphate-citrate, pH = 4.0] for 30 min at room temperature. The experiment was repeated three times, independently.

### Assessment of the serum interference in the anti-PEG bioparticle-based sandwich ELISA

Saline, saline containing 2% (wt/vol) skim milk or saline containing 2.5% (v/v) or 10% (v/v) mouse or human serum were used as diluents for LipoDox and PEG-QD (n = 3 per group). The anti-PEG bioparticle-based sandwich ELISA was then performed as described above; saline was used as the wash buffer and saline containing 2% (wt/vol) skim milk was used as the diluent for secondary and tertiary reagents. The experiment was repeated three times, independently.

### Pharmacokinetics of Nano-X-^111^In in mice

Female BALB/c mice (n = 8) was intravenously injected with Nano-X-^111^In. The mouse blood was harvested at different times by use of a heparinized capillary tube. The concentration of Nano-X-^111^In was measured by anti-PEG bioparticle-based ELISA and a gamma counter for 50- and 250-fold diluted serum. The radioactivity of weighed serum was counted on a Wallac 1470 Wizard gamma counter (Perkin-Elmer). The serum half-life of Nano-X-^111^In was estimated by fitting the data to a one-phase exponential decay model with Prism 6 software.

### Statistical Analysis

The detection limit of the anti-PEG bioparticle-based sandwich ELISA in Figs 3, 4, 5 was defined as the lowest concentration of PEGylated nanoparticles that produced a statistically higher signal than the signal produced from the blank. Statistical significance was calculated using GraphPad Prism 6.0 with student t-test. In Fig. 3, the statistical difference of paraformaldehyde fixation and non-fixation groups was analyzed by independent t-test. In Fig. 6, the statistical difference of the anti-PEG bioparticle-based ELISA and the gamma counter to assess the pharmacokinetics of Nano-X-^111^In was analyzed by independent t-test. Data were considered statistically different as P values < 0.05. In Fig. 5, the statistical comparison between control group (saline containing 2% skim milk) and test groups was performed by multiple t-test. *P value < 0.05; **P value < 0.0001 as compared to control group. The serum half-life of Nano-X-^111^In was calculated using GraphPad Prism 6.0 with one-phase exponential decay model.

## Additional Information

**How to cite this article**: Hsieh, Y.-C. *et al*. Using anti-poly(ethylene glycol) bioparticles for the quantitation of PEGylated nanoparticles. *Sci. Rep.* **6**, 39119; doi: 10.1038/srep39119 (2016).

**Publisher's note:** Springer Nature remains neutral with regard to jurisdictional claims in published maps and institutional affiliations.

## Figures and Tables

**Figure 1:**
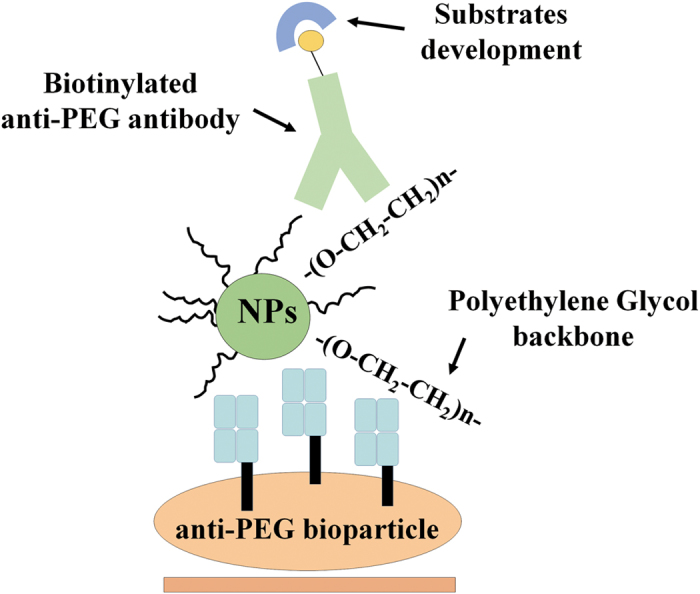
Anti-PEG bioparticle-based ELISA systems. Schematic representation of the anti-PEG-bioparticles. The anti-PEG bioparticles are derived from the BALB/c 3T3 cells stably expressing anti-PEG antibody Fab on the cell membrane. The sandwich ELISA is generated by PEG-bioparticle and the detective biotinylated anti-PEG antibody, which can estimate the PEGylated nanoparticles (NPs).

## References

[b1] Duggan S. T. & Keating G. M. Pegylated liposomal doxorubicin: a review of its use in metastatic breast cancer, ovarian cancer, multiple myeloma and AIDS-related Kaposi's sarcoma. Drugs 71, 2531–2558, doi: 10.2165/11207510-000000000-00000 (2011). 22141391

[b2] Chao T. C. . A dose-escalating pilot study of sterically stabilized, pegylated liposomal doxorubicin (Lipo-Dox) in patients with metastatic breast cancer. Cancer Invest 21, 837–847 (2003). 14735687 10.1081/cnv-120025086

[b3] Liu C. H. . Peginterferon plus Ribavirin for HIV-infected Patients with Treatment-Naive Acute or Chronic HCV Infection in Taiwan: A Prospective Cohort Study. Sci Rep 5, 17410, doi: 10.1038/srep17410 (2015). 26616669 PMC4663763

[b4] Liu C. H. . Peginterferon plus weight-based ribavirin for treatment-naive hepatitis C virus genotype 2 patients not achieving rapid virologic response: a randomized trial. Sci Rep 5, 11710, doi: 10.1038/srep11710 (2015). 26130141 PMC4486927

[b5] Zeuzem S. Interferon-based therapy for chronic hepatitis C: current and future perspectives. Nat Clin Pract Gastroenterol Hepatol 5, 610–622, doi: 10.1038/ncpgasthep1274 (2008). 18838975

[b6] Plummer R. . A Phase I clinical study of cisplatin-incorporated polymeric micelles (NC-6004) in patients with solid tumours. British journal of cancer 104, 593–598, doi: 10.1038/bjc.2011.6 (2011). 21285987 PMC3049602

[b7] Oerlemans C. . Polymeric micelles in anticancer therapy: targeting, imaging and triggered release. Pharmaceutical research 27, 2569–2589, doi: 10.1007/s11095-010-0233-4 (2010). 20725771 PMC2982955

[b8] Walling M. A., Novak J. A. & Shepard J. R. Quantum dots for live cell and *in vivo* imaging. International journal of molecular sciences 10, 441–491, doi: 10.3390/ijms10020441 (2009). 19333416 PMC2660663

[b9] Wang Y. X. Superparamagnetic iron oxide based MRI contrast agents: Current status of clinical application. Quantitative imaging in medicine and surgery 1, 35–40, doi: 10.3978/j.issn.2223-4292.2011.08.03 (2011). 23256052 PMC3496483

[b10] Wei G., Xiao S., Si D. & Liu C. Improved HPLC method for doxorubicin quantification in rat plasma to study the pharmacokinetics of micelle-encapsulated and liposome-encapsulated doxorubicin formulations. Biomed Chromatogr 22, 1252–1258, doi: 10.1002/bmc.1054 (2008). 18651589

[b11] Myllynen P. K. . Kinetics of gold nanoparticles in the human placenta. Reprod Toxicol 26, 130–137, doi: 10.1016/j.reprotox.2008.06.008 (2008). 18638543

[b12] Crayton S. H., Elias D. R., Al Zaki A., Cheng Z. & Tsourkas A. ICP-MS analysis of lanthanide-doped nanoparticles as a non-radiative, multiplex approach to quantify biodistribution and blood clearance. Biomaterials 33, 1509–1519, doi: 10.1016/j.biomaterials.2011.10.077 (2012). 22100983 PMC3237748

[b13] Chang H. I. & Yeh M. K. Clinical development of liposome-based drugs: formulation, characterization, and therapeutic efficacy. International journal of nanomedicine 7, 49–60, doi: 10.2147/IJN.S26766 (2012). 22275822 PMC3260950

[b14] Ryan S. M., Mantovani G., Wang X., Haddleton D. M. & Brayden D. J. Advances in PEGylation of important biotech molecules: delivery aspects. Expert opinion on drug delivery 5, 371–383, doi: 10.1517/17425247.5.4.371 (2008). 18426380

[b15] Cheng T. L., Chuang K. H., Chen B. M. & Roffler S. R. Analytical measurement of PEGylated molecules. Bioconjug Chem 23, 881–899, doi: 10.1021/bc200478w (2012). 22242549

[b16] Lankveld D. P. . Blood clearance and tissue distribution of PEGylated and non-PEGylated gold nanorods after intravenous administration in rats. Nanomedicine (Lond) 6, 339–349, doi: 10.2217/nnm.10.122 (2011). 21385136

[b17] Varna M. . &lt;i&gt;*In vivo*&lt;/i&gt; Distribution of Inorganic Nanoparticles in Preclinical Models. Journal of Biomaterials and Nanobiotechnology Vol.03No.02, 11, doi: 10.4236/jbnb.2012.322033 (2012).

[b18] Chuang K.-H. . Development of an Anti-Methoxy Poly(ethylene glycol) (α-mPEG) Cell-Based Capture System to Measure mPEG and mPEGylated Molecules. Macromolecules 47, 6880–6888, doi: 10.1021/ma501156r (2014).

[b19] Su Y. C., Chen B. M., Chuang K. H., Cheng T. L. & Roffler S. R. Sensitive quantification of PEGylated compounds by second-generation anti-poly(ethylene glycol) monoclonal antibodies. Bioconjug Chem 21, 1264–1270, doi: 10.1021/bc100067t (2010). 20536171

[b20] Tate J. & Ward G. Interferences in immunoassay. The Clinical biochemist. Reviews/Australian Association of Clinical Biochemists 25, 105–120 (2004). PMC1904417 18458713

[b21] Selby C. Interference in immunoassay. Annals of clinical biochemistry 36 (Pt 6), 704–721 (1999). 10586307 10.1177/000456329903600603

[b22] Bylda C., Thiele R., Kobold U. & Volmer D. A. Recent advances in sample preparation techniques to overcome difficulties encountered during quantitative analysis of small molecules from biofluids using LC-MS/MS. The Analyst 139, 2265–2276, doi: 10.1039/c4an00094c (2014). 24633191

[b23] Chambers E., Wagrowski-Diehl D. M., Lu Z. & Mazzeo J. R. Systematic and comprehensive strategy for reducing matrix effects in LC/MS/MS analyses. Journal of chromatography. B, Analytical technologies in the biomedical and life sciences 852, 22–34, doi: 10.1016/j.jchromb.2006.12.030 (2007). 17236825

[b24] Remsberg C. M. . Pharmacokinetic Evaluation of a DSPE-PEG2000 Micellar Formulation of Ridaforolimus in Rat. Pharmaceutics 5, 81–93, doi: 10.3390/pharmaceutics5010081 (2012). 24300398 PMC3834941

[b25] Cheng T. C. . Sensitivity of PEGylated interferon detection by anti-polyethylene glycol (PEG) antibodies depends on PEG length. Bioconjug Chem 24, 1408–1413, doi: 10.1021/bc3006144 (2013). 23837865

[b26] Tsai H. J., Chi L. A. & Yu A. L. Monoclonal antibodies targeting the synthetic peptide corresponding to the polybasic cleavage site on H5N1 influenza hemagglutinin. Journal of biomedical science 19, 37, doi: 10.1186/1423-0127-19-37 (2012). 22471562 PMC3366877

[b27] Jackson L. R., Trudel L. J., Fox J. G. & Lipman N. S. Monoclonal antibody production in murine ascites. I. Clinical and pathologic features. Laboratory animal science 49, 70–80 (1999). 10090098

[b28] Bodor G. S., Porter S., Landt Y. & Ladenson J. H. Development of monoclonal antibodies for an assay of cardiac troponin-I and preliminary results in suspected cases of myocardial infarction. Clinical chemistry 38, 2203–2214 (1992). 1424112

[b29] Marx U. E. M., Fischer R., Gruber F. P., Hansson U., Heuer J., de Leeuw W. A., Logtenberg T., Merz W., Portetelle D., Romette J. L. & Straughan D. W. Monoclonal antibody production: The report and recommendations of ECVAM Workshop 23. ATLA. 25 (1997).

[b30] UKCCCR guidelines for the welfare of animals in experimental neoplasia. British journal of cancer 58, 109–113 (1988). 3166888 10.1038/bjc.1988.174 PMC2246499

[b31] Kuhlmann I., Kurth W. & Ruhdel I. Monoclonal antibodies: *in vivo* and *in vitro* production on a laboratory scale, with consideration of the legal aspects of animal protection. ATLA. 17, 73–82 (1985). 11208272

[b32] Hendriksen C. F. & de Leeuw W. Production of monoclonal antibodies by the ascites method in laboratory animals. Research in immunology 149, 535–542 (1998). 9835414

[b33] Mariani E. . Commercial serum-free media: hybridoma growth and monoclonal antibody production. Journal of immunological methods 145, 175–183 (1991). 1765649 10.1016/0022-1759(91)90324-9

[b34] Glassy M. C., Tharakan J. P. & Chau P. C. Serum-free media in hybridoma culture and monoclonal antibody production. Biotechnology and bioengineering 32, 1015–1028, doi: 10.1002/bit.260320809 (1988). 18587819

[b35] Chuang K. H. . Measurement of poly(ethylene glycol) by cell-based anti-poly(ethylene glycol) ELISA. Anal Chem 82, 2355–2362, doi: 10.1021/ac902548m (2010). 20178318

[b36] Suk J. S., Xu Q., Kim N., Hanes J. & Ensign L. M. PEGylation as a strategy for improving nanoparticle-based drug and gene delivery. Adv Drug Deliv Rev 99, 28–51, doi: 10.1016/j.addr.2015.09.012 (2016). 26456916 PMC4798869

[b37] Chuang K. H. . Endocytosis of PEGylated agents enhances cancer imaging and anticancer efficacy. Mol Cancer Ther 9, 1903–1912, doi: 10.1158/1535-7163.MCT-09-0899 (2010). 20501805

[b38] Chow T. H. . Diagnostic and therapeutic evaluation of 111In-vinorelbine-liposomes in a human colorectal carcinoma HT-29/luc-bearing animal model. Nucl Med Biol 35, 623–634, doi: 10.1016/j.nucmedbio.2008.04.001 (2008). 18589307

